# Stiff-person syndrome with concurrent Graves disease: a rare autoimmune overlap

**DOI:** 10.1210/jcemcr/luag058

**Published:** 2026-03-24

**Authors:** Melanie Natasha Rayan, Yara Alshawabkeh, Varsha Irvathraya, Benjamin O’Donnell

**Affiliations:** Division of Endocrinology, Diabetes and Metabolism, Ohio State University Medical Center, Columbus, OH 43210, USA; University of Central Florida College of Medicine, Gainesville, FL 32605, USA; Ohio State University College of Medicine, Columbus, OH 43210, USA; Division of Endocrinology, Diabetes and Metabolism, Ohio State University Medical Center, Columbus, OH 43210, USA

**Keywords:** stiff-person syndrome, GAD encephalitis, Graves disease, GAD65

## Abstract

Stiff-person syndrome (SPS) is an autoimmune neurologic disorder characterized by progressive rigidity and spasms, often linked to antiglutamic acid decarboxylase 65 antibodies. While SPS has established associations with autoimmune diseases like type 1 diabetes and celiac disease, co-occurrence with hyperthyroidism, particularly Graves disease (GD), is rare. We present the case of a 34-year-old woman with paranoia, hallucinations, and altered mental status. Labs revealed thyrotoxicosis, with positive thyroid-stimulating immunoglobulin and thyrotropin receptor antibodies, confirming GD. Despite antithyroid treatment, her neuropsychiatric symptoms progressed. Though the neurologic workup was unremarkable, cerebrospinal fluid studies revealed oligoclonal bands with markedly elevated antiglutamic acid decarboxylase 65 antibodies, consistent with autoimmune encephalitis within the SPS spectrum. Therapeutic plasma exchange and rituximab resulted in rapid clinical improvement. At 6 weeks, thyroid-stimulating immunoglobulin and thyrotropin receptor antibody levels normalized, and she remained stable off antithyroid therapy, suggesting resolution of GD. This case illustrates the rare coexistence of SPS and GD.

## Introduction

Stiff-person syndrome (SPS) is a rare autoimmune neurological disorder characterized by progressive muscle stiffness, exaggerated lumbar lordosis, and frequent falls. Muscle spasms are often precipitated by sudden sensory stimuli, emotional stress, or voluntary movements [1]. Stiff-person syndrome predominantly affects women (∼70% of cases) and typically presents between ages 20 and 60, with a median age of 40 [2]. Despite growing recognition, limited awareness of complex presentations often leads to delayed diagnosis.

Stiff-person syndrome arises from impaired gamma-aminobutyric acid (GABA)-mediated inhibition due to autoantibodies against glutamic acid decarboxylase 65 (GAD65). Up to 70% of patients with GAD65 antibodies have at least 1 additional autoimmune disorder, most commonly type 1 diabetes mellitus and autoimmune thyroiditis [3]. While thyroid autoimmunity, especially Hashimoto thyroiditis, is well-documented in SPS [4, 5], coexisting Graves disease (GD) is exceedingly rare and limited to case reports [6-9].

## Case presentation

A 34-year-old woman with a history of depression, cannabis use disorder, and suspected cyclic vomiting syndrome was transferred from a psychiatric facility to our emergency department for persistent tachycardia, agitation, and profound fatigue. Three weeks prior, she had been admitted for acute-onset paranoia, auditory hallucinations, and behavioral disturbances, including fear of contamination, present for several weeks and accompanied by a 15-pound weight loss. She was started on aripiprazole 10 mg daily at the psychiatric facility.

On arrival to the emergency department, the patient appeared confused and reported visual hallucinations. Vitals showed tachycardia (heart rate 100-120 bpm) with otherwise stable vital signs. She was tremulous, with a mild goiter on examination. Laboratory evaluation revealed thyrotoxicosis with thyroid-stimulating hormone (TSH) <0.008 μIU/mL (<0.008 mIU/L; reference range, RR: 0.4-4.0 μIU/mL; 0.4-4.0 mIU/L), free thyroxine (T4) 4.13 ng/dL (53.2 pmol/L; [RR]: 0.8-1.8 ng/dL; 10.3-23.2 pmol/L), and total triiodothyronine (T3) 4.98 ng/mL (7.64 nmol/L; RR: 0.8-2.0 ng/mL; 1.23-3.07 nmol/L). A TSH result 1 year prior had been normal. Mild transaminitis was noted. She had no personal or family history of autoimmune disease.

**Figure 1:**
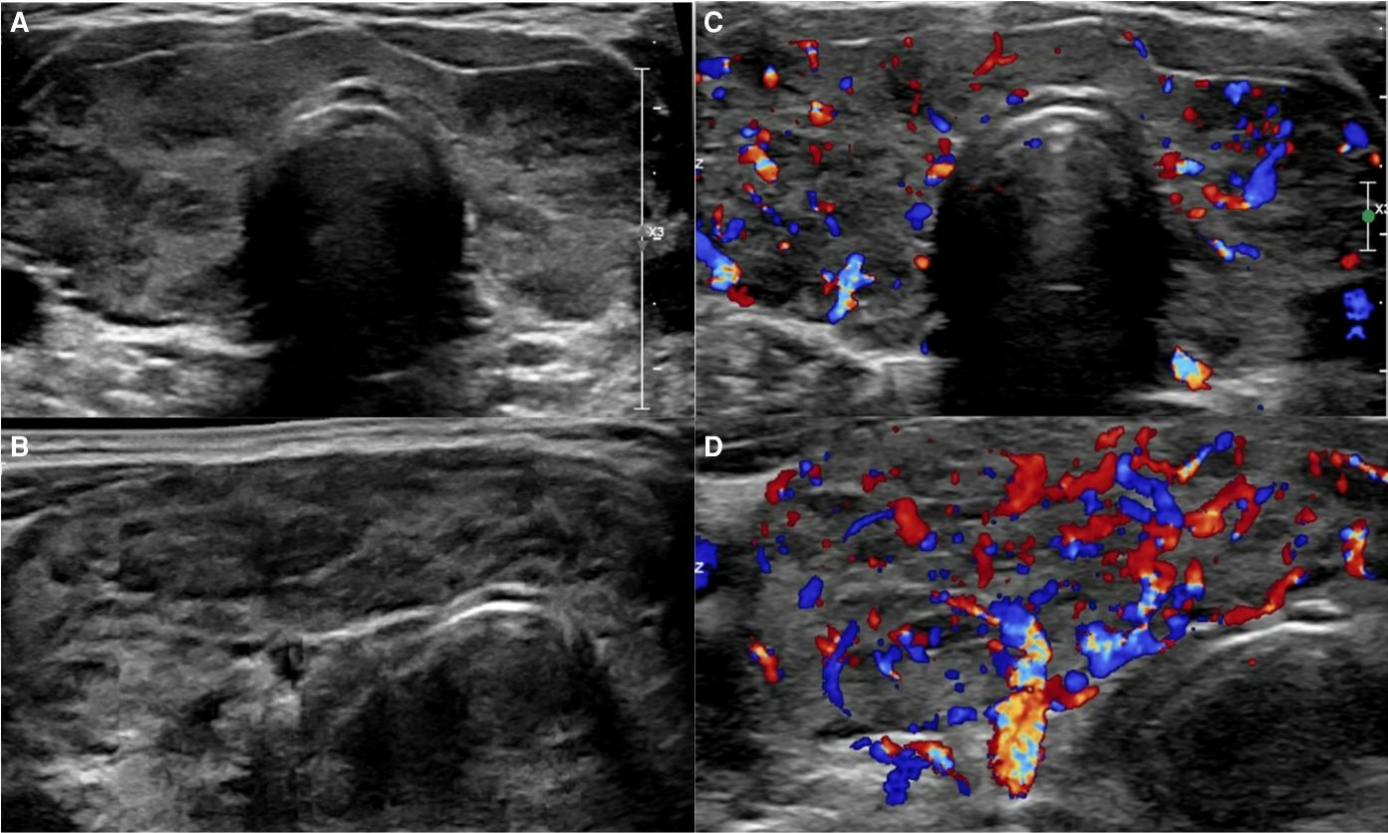
Thyroid ultrasound with color-flow Doppler. (A, C) Gray-scale images show diffuse thyroid enlargement and heterogeneous echotexture. (B, D) Color Doppler images demonstrate diffuse symmetric hypervascularity consistent with GD.

Endocrinology was consulted, and the diagnosis of GD was made based on clinical features, including diffuse goiter, tachycardia, tremor, and weight loss, along with elevated thyroid-stimulating immunoglobulin (TSI) 3.0 IU/L (RR: <0.55 IU/L), thyrotropin receptor antibodies (TRAb) 4.05 IU/L (RR: 0-0.9 IU/L). Antithyroid peroxidase (TPO) antibody was also elevated to 300.2 IU/mL (RR: <35 IU/mL), consistent with underlying thyroid autoimmunity. Thyroid ultrasound revealed a diffusely enlarged, heterogeneous, and hypervascular gland confirmed by color-flow Doppler (Fig. 1). The patient began methimazole 20 mg daily and propranolol 25 mg twice daily.

Broad laboratory testing (complete blood count, comprehensive metabolic panel, vitamin B12, folate, beta human chorionic gonadotropin (β-hCG), erythrocyte sedimentation rate, and intact parathyroid hormone) was unrevealing, except for elevated creatinine kinase 561 U/L (RR: 20-200 U/L). Computed tomography of the head and electroencephalogram and magnetic resonance imaging of the brain were performed to exclude structural or acute intracranial pathology as a cause of her encephalopathy, and all were normal. Computed tomography of the abdomen/pelvis done to exclude other systemic causes was normal. Cerebrospinal fluid (CSF) analysis revealed mild lymphocytic pleocytosis with 9 white blood cells/μL (9 × 10^6^/L; RR: 0-5/μL; 0-5 × 10^6^/L), glucose of 67 mg/dL (3.7 mmol/L; RR: 50-80 mg/dL; 2.8-4.4 mmol/L), and protein of 23 mg/dL (0.23 g/L; RR: 15-45 mg/dL; 0.15-0.45 g/L). Cerebrospinal fluid autoimmune antibody testing was ordered, including GAD65 antibody, *N*-methyl-D-aspartate receptor antibody, and GABA B receptor (GABA-B-R) antibody, as well as a panel of paraneoplastic markers.

Despite resolution of tachycardia, neuropsychiatric symptoms persisted, prompting replacement of aripiprazole with risperidone. The patient was discharged to psychiatry for ongoing care after 10 days. However, the following day, she was found slumped in her seat with drooling, unresponsiveness, and extremity tremors, prompting immediate readmission.

## Diagnostic assessment

Upon readmission, neurological examination revealed nonrhythmic left-predominant tremors, bilateral upper extremity stiffness, and hyperreflexia. Electroencephalogram showed mild diffuse encephalopathy; magnetic resonance imaging remained normal. Repeat thyroid function testing showed improvement, with a free T4 2.27 ng/dL (29.2 pmol/L) and total T3 2.71 ng/mL (4.16 nmol/L). Pending CSF results returned positive for 13 oligoclonal bands in CSF (absent in serum), suggestive of intrathecal antibody production. Cerebrospinal fluid GAD65 antibodies were markedly elevated at 183 IU/mL (RR: <5 IU/mL), and serum GAD65 was 1600 IU/mL (RR: <5 IU/mL), confirming GAD65 antibody–mediated autoimmune encephalitis. All other autoimmune CSF antibodies returned negative. Hashimoto encephalopathy was considered, but high intrathecal GAD65 antibodies with CSF oligoclonal bands confirmed autoimmune encephalitis in the SPS spectrum.

## Treatment

She received intravenous (IV) methylprednisolone 1 g daily for suspected autoimmune encephalitis. Once the diagnosis was confirmed, she received 5 sessions of therapeutic plasma exchange and started baclofen 5 mg thrice daily for rigidity. She improved steadily, becoming interactive, alert, and following commands. Rigidity, myoclonus, and hyperreflexia diminished.

Methimazole was tapered to 10 mg, then 5 mg, and discontinued after thyroid stabilization in response to improving thyroid labs: free T4 of 0.87 ng/dL (11.2 pmol/L) and total T3 of 0.46 ng/dL (0.71 nmol/L), TSH 0.010 μIU/mL (0.010 mIU/L), free T4 of 0.64 ng/dL (8.2 pmol/L), and total T3 0.28 ng/mL (0.43 nmol/L; Table 1). Despite marked improvement with plasma exchange, persistently high intrathecal GAD65 titers prompted a single 1000 mg rituximab dose before discharge.

**Table 1:** Thyroid function tests timeline with methimazole doses

Date	TSH (μIU/mL [mIU/L]) (RR: 0.4-4.0 μIU/mL; 0.4-4.0 mIU/L)	Free T4 (ng/dL [pmol/L]) (RR: 0.8-1.8 ng/dL; 10.3-23.2 pmol/L)	Total T3 (ng/mL [nmol/L]) (RR: 0.8-2.0 ng/mL; 1.23-3.07 nmol/L)	Methimazole dose (mg/day)
10/27	<0.008 (<0.008 mIU/L)	4.13 (53.2 pmol/L)	4.98 (7.64 nmol/L)	20
11/11	<0.008 (<0.008 mIU/L)	2.27 (29.2 pmol/L)	2.71 (4.16 nmol/L)	20
11/17	<0.008 (<0.008 mIU/L)	0.87 (11.2 pmol/L)	0.46 (0.71 nmol/L)	10
11/20	0.008 (0.008 mIU/L)	0.81 (10.4 pmol/L)	0.53 (0.81 nmol/L)	5
11/24	0.010 (0.010 mIU/L)	0.64 (8.2 pmol/L)	0.28 (0.43 nmol/L)	Stopped
11/27	0.008 (0.008 mIU/L)	0.63 (8.1 pmol/L)	0.40 (0.61 nmol/L)	Stopped
11/30	0.008 (0.008 mIU/L)	0.72 (9.3 pmol/L)	0.64 (0.98 nmol/L)	Stopped
1/29 (FU)	0.583 (0.583 mIU/L)	0.66 (8.5 pmol/L)	0.81 (1.24 nmol/L)	Stopped

Abbreviations: FU, follow-up; RR, reference range; T3, triiodothyronine; T4, thyroxine; TSH, thyroid-stimulating hormone.

## Outcome and follow-up

At 6-week outpatient follow-up, thyroid studies demonstrated euthyroidism with a TSH of 0.583 μIU/mL (0.583 mIU/L), free T4 of 0.66 ng/dL (8.5 pmol/L), and total T3 of 0.81 ng/mL (1.24 nmol/L). Thyrotropin receptor antibodies and TSI were negative at follow-up, supporting remission of GD and arguing against transient thyroiditis or hashitoxicosis. She continues rituximab infusions every 6 months to sustain the improvement achieved after plasma exchange, lower the chance of relapse, and avoid the risks of prolonged corticosteroid use.

## Discussion

This case describes a rare overlap of SPS and GD, presenting with prominent neuropsychiatric symptoms and thyrotoxicosis. The patient's acute onset of hallucinations, paranoia, tremors, and weight loss, without prior autoimmune or psychiatric history, created a diagnostic challenge. Thyroid storm was considered, but the patient remained afebrile, in sinus rhythm, and showed no evidence of heart failure or gastrointestinal/hepatic dysfunction. Thyroid hormone levels were already declining on methimazole when neuropsychiatric symptoms progressed. Oligoclonal bands and high intrathecal GAD65 antibodies confirmed SPS-spectrum autoimmune encephalitis. GAD65 antibodies are not known to exert a direct pathogenic effect on thyroid tissue; their presence in this case more likely reflects a shared immunogenetic background. In SPS, intrathecal GAD65 antibody synthesis with B-cell activity impairs GABAergic inhibition, causing cortical and spinal hyperexcitability [10]. Both SPS and GD share immunogenetic susceptibilities, particularly human leukocyte antigen (HLA)-DR and HLA-DQ haplotypes, which predispose to antigen presentation defects, loss of tolerance, and clustering of autoimmunity [10]. The concurrent remission of neurological and thyroid disease following plasma exchange and rituximab supports a common immunopathogenic pathway, wherein B-cell-directed immune modulation can ameliorate distinct but related autoimmune syndromes.

Notably, therapeutic plasma exchange led to rapid resolution of psychiatric and motor symptoms with normalization of thyroid function. The patient was able to discontinue methimazole entirely, and follow-up testing showed negative TRAb and TSI titers, confirming biochemical remission of GD. In GD alone, plasma exchange typically provides only transient biochemical improvement and serves as a bridge to definitive therapy for severe thyrotoxicosis. In contrast, the sustained remission observed in our patient, with negative TRAb and TSI off antithyroid therapy, suggests that rituximab contributed to durable modulation of autoimmune activity by selectively reducing stimulatory TRAb through rapid anti-CD20-mediated depletion of activated and memory B cells, providing a biologically plausible mechanism for the observed remission [11]. This finding indicates that targeting GAD65-mediated autoimmunity effectively addressed both the neurological and thyroid manifestations, supporting the hypothesis of a shared autoimmune process. Although spontaneous remission of GD can occur, it is usually gradual and follows prolonged antithyroid therapy. Our patient experienced rapid improvement following plasma exchange and rituximab, making spontaneous recovery an unlikely explanation.

While classically considered a neurological disorder, SPS frequently presents with features transcending motor dysfunction. Autonomic involvement is common and may manifest as episodic tachycardia, diaphoresis, hypertension, pupillary abnormalities, hyperpyrexia, or autonomic crises, which can culminate in sudden death [12]. Neuropsychiatric symptoms are common in SPS but are underrecognized. Anxiety disorders, which may include agoraphobia or basophobia, occur in 50% to 56% and often relate to spasms triggered by emotional stimuli [13] and 40% to 45% of patients experience depression. Less common psychiatric conditions include eating disorders and alcohol abuse [14]. In some cases, psychiatric symptoms such as hallucinations, paranoia, or confusion predominate, leading to misdiagnoses as a primary psychiatric illness and delaying appropriate evaluation. This diagnostic pitfall was evident, as the initial presentation was dominated by psychiatric symptoms. The clinical picture was further complicated by the patient's history of cannabis use disorder. Cannabis exposure may worsen anxiety, paranoia, and psychotic-like symptoms, including hallucinations [15]. While her psychiatric presentation was ultimately explained by autoimmune encephalitis, the coexisting cannabis use history may have contributed to diagnostic uncertainty and delayed recognition of the underlying neurological process.

Autoimmune comorbidities are common in SPS, with around 70% of GAD65-positive patients having at least one other autoimmune condition such as vitiligo, celiac disease, pernicious anemia, type 1 diabetes, or Hashimoto thyroiditis [16]. These conditions may precede or follow neurological manifestations of SPS and reflect a shared underlying autoimmune mechanism. Among thyroid autoimmunity, Hashimoto thyroiditis is the most commonly reported association, with several large case series supporting its co-occurrence with SPS [2, 5]. A retrospective study of 205 patients found that 30% of individuals with SPS were positive for TPO antibodies [17].

Graves disease, conversely, is a less well-documented clinically significant endocrine manifestation of SPS. In a 2012 systematic review of 121 patients with GAD antibody–associated syndromes, Martinez-Hernandez et al assessed thyroid autoimmunity using antithyroglobulin and anti-TPO antibodies but did not evaluate TSI or TRAb [18]. Few cases exist in the literature, and fewer confirm the presence of thyroid antibodies. The first reported case, in 1961, described a 44-year-old man with progressive stiffness, spasms, gait disturbance, and dysphagia, alongside hyperthyroid symptoms including weight loss, palpitations, and pretibial myxedema [9]. He was diagnosed with GD based on elevated TRAb and radioactive iodine uptake. Despite antithyroid therapy, neuromuscular symptoms persisted. In 1990, Solimena et al reported GD in 4 of 20 GAD65-positive SPS patients, with none among the GAD65-negative group. Although TPO and thyroglobulin antibodies were tested, TRAb and TSI were not, and diagnostic criteria for GD were not specified [19]. A 2005 case described a 52-year-old woman with SPS and antibody-positive GD who showed significant improvement in neurologic and thyroid symptoms following immunosuppressive therapy, suggesting an autoimmune link [7]. In 2007, Chia et al reported a middle-aged woman initially treated for GD with limited response who later developed stroke-like symptoms and was diagnosed with SPS [20]. Although TRAb was not tested, positive TSI and TPO antibodies supported autoimmunity. Immunotherapy improved both neurological and thyroid symptoms, and her thyroid function remained stable on low-dose carbimazole, though TSI levels remained elevated. In 2016, Medeiros et al reported a 9-year-old girl with falls, gait abnormalities, weight loss, and a tonic-clonic seizure. She was diagnosed with GD and elevated GAD65 antibodies, improving significantly following IV immunoglobulin [8].

In our case, the patient's clinical course followed an unfortunately common trajectory in SPS. She was initially admitted to a psychiatric facility with paranoia and hallucinations and then transferred to our hospital, where she was found to have thyrotoxicosis and was diagnosed with GD. Although appropriate treatment was initiated, this diagnosis did not fully explain the severity of her neuropsychiatric and motor symptoms. This case underscores the need to recognize the broad clinical spectrum of GAD65-associated autoimmunity and highlights the importance of early, multidisciplinary evaluation in patients with overlapping autoimmune, psychiatric, and neurologic symptoms. Although rare, SPS symptoms can mimic common conditions such as anxiety, depression, and thyroid or pancreatic autoimmunity. Clinicians should maintain a high index of suspicion, as early recognition enables targeted therapy with potentially broad therapeutic benefits beyond the primary neurological disorder.

## Learning points

- SPS is a rare autoimmune disorder caused by GAD65 autoantibodies, frequently coexisting with other autoimmune conditions, most commonly type 1 diabetes and thyroid autoimmunity.
- Symptoms include progressive stiffness and spasms, often triggered by sensory stimuli or stress. Neuropsychiatric symptoms, including anxiety and depression, are common in SPS but underrecognized, leading to misdiagnosis as primary psychiatric illness.
- Coexistence of SPS and GD is exceptionally rare but may respond to therapeutic plasma exchange, with potential resolution of both neurological and thyroid autoimmunity.
- Multidisciplinary collaboration between neurology, endocrinology, and psychiatry is essential for optimal management of complex autoimmune presentations.

## Contributors

All authors made individual contributions to authorship. M.N.R. and B.O. were involved in the diagnosis and management of this patient. M.N.R., V.I., and Y.A. were involved in the manuscript submission. Y.A. and V.I. were involved in the preparation of ultrasound images. All authors reviewed and approved the final draft.

## Data Availability

Data sharing is not applicable to this article, as no datasets were generated or analyzed during the current study.
